# Effects of Coronary Ischemia-Reperfusion in a Rat Model of Early Overnutrition. Role of Angiotensin Receptors

**DOI:** 10.1371/journal.pone.0054984

**Published:** 2013-02-01

**Authors:** Miriam Granado, Nuria Fernández, Luis Monge, Juan Carlos Figueras, Gonzalo Carreño-Tarragona, Sara Amor, Angel Luis García-Villalón

**Affiliations:** 1 Department of Physiology, Faculty of Medicine, Universidad Autónoma de Madrid, Madrid, Spain; 2 CIBER Fisiopatología de Obesidad y Nutrición, Instituto de Salud Carlos III, Madrid, Spain; John Hunter Hospital, Australia

## Abstract

**Background:**

Obesity during childhood has dramatically increased worldwide in the last decades. Environmental factors acting early in life, including nutrition, play an important role in the pathogenesis of obesity and cardiovascular diseases in adulthood.

**Aims:**

To analyze the effects of early overfeeding on the heart and coronary circulation, the effect of ischemia-reperfusion (I/R) and the role of the renin-angiotensin system (RAS) were studied in isolated hearts from control and overfed rats during lactation.

**Methods and Results:**

On the day of birth litters were adjusted to twelve pups per mother (control) or to three pups per mother (overfed). At weaning (21 days) the rats were killed and the heart perfused in a Langendorff system and subjected to 30 min of ischemia followed by 15 min of reperfusion. The contractility (left developed intraventricular pressure) was lower in the hearts from overfed rats, and was reduced by I/R in hearts from control but not from overfed rats. I/R also reduced the coronary vasoconstriction to angiotensin II more in hearts from control than from overfed rats, and the vasodilatation to bradykinin similarly in both experimental groups. The expression of both angiotensin *AGTRa* and *AGTR2* receptors was increased in the myocardium of overfed rats, and I/R increased the expression of both receptors in control rats but reduced it in overfed rats. The expression of apoptotic and antiapoptotic markers was increased in hearts of overfed rats compared with control, and further increased by I/R.

**Conclusions:**

These results suggest that both overfeeding and I/R impair cardiac and coronary function due, at least in part, to activation of the angiotensin pathway. However, overfeeding may reduce the impairment of ventricular contractility by I/R.

## Introduction

Cardiovascular diseases are the leading cause of death in the developed countries, and one of the main risk factors for cardiovascular mortality is obesity. The incidence of obesity is increasing at a rapid rate, particularly in children and adolescents [1]. Moreover, being overweight at a young age predisposes to adult obesity [2]and induces irreversible changes in the cardiovascular system leading to impairment of cardiac and coronary function in the adult [3] increasing the risk of suffering coronary disease [4], [5]later in life. Likewise, in experimental animals perinatal overnutrition induced by either maternal obesity [6] or by postnatal overfeeding [7] has been reported to induce long-term effects in metabolism and cardiovascular function [8] possible due to changes in postnatal leptin levels [9].

Recent studies suggest that angiotensin II may be one of the factors promoting cardiovascular disease in the obese. Angiotensin II is produced by enzymatic cleavage of the precursor angiotensinogen by renin and by angiotensin-converting enzyme (ACE), and exerts its effects in the tissues through angiotensin receptors type1 (*AGTRa*) and type 2 (*AGTR2*). These components of the renin-angiotensin system are present in visceral and subcutaneous adipose tissue [10], and are increased in obesity [11]. There is a positive correlation between obesity and angiotensinogen expression in adipose tissue both in humans [12], [13] and in rats [14], [15]. In addition renin *(REN)*, *ACE* and *AGTRa* expression are also increased in adipose tissue from obese subjects [16]. Moreover, there is evidence that RAS activation is correlated with cardiovascular risk factors and cardiovascular disease [17]. Angiotensin II is known to promote oxidative stress, which may lead to activation of inflammatory [18]and apoptotic [19]pathways. These effects of the RAS may be particularly important during young age, as angiotensin also plays a role in kidney and vascular development [20].

Therefore, the aim of the present study is to analyze the effects of early overfeeding on the heart and coronary circulation, comparing both the effect of ischemia-reperfusion (I/R) and the role of the RAS in control and overfed rats. For this purpose we have used an experimental model of early overnutrition induced by litter reduction. This experimental model is reported to induce an increase in food intake and weight gain during lactation that lasts after weaning [21]. The increase in body weight and body fat is also accompanied with hyperleptinemia [22] and hyperinsulinemia [23]. This experimental model may reproduce several characteristics of childhood obesity in humans.

## Materials and Methods

### Animals

#### Ethics statement

Sprague-Dawley rats were used for these studies (Harlan interfauna Ibérica S.A., Barcelona, Spain). All the experiments were conducted in accordance with the US National Institutes of Health Guide for the Care and Use of Laboratory Animals (NIH Publication No. 85-23, revised 1996) and in compliance with all relevant laws and regulations. The use of these animals was also approved by the Institute’s Animal Care and Use Committee (Comité de Ética de la Investigación, Universidad Autónoma de Madrid).

After mating and pregnancy was confirmed, dams were housed individually and fed ad libitum until the end of pregnancy. On the day of birth 12 litters were adjusted to twelve pups per mother (control) and 16 litters were adjusted to three pups per mother (overfed).

### Heart Perfusion

**Figure 1:**
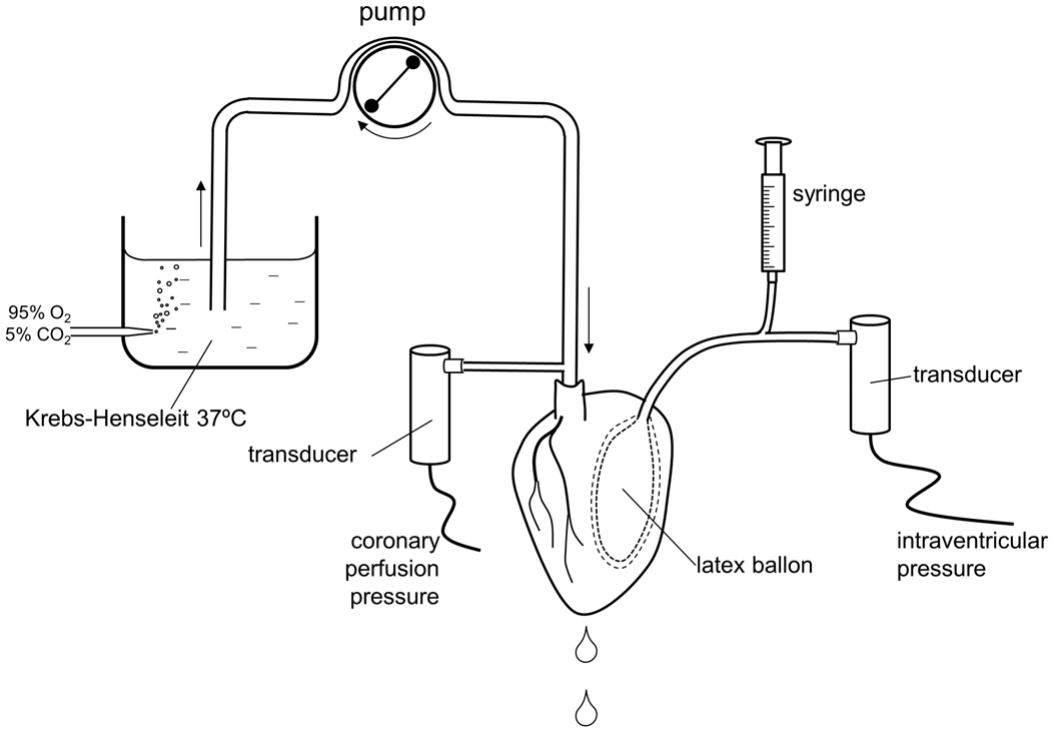
Schematic representation of the experimental set up used to measure coronary perfusion pressure and intraventricular pressure in the perfused rat heart.

The hearts were removed from the rats under anaesthesia with i.p. sodium pentobarbital (200 mg/kg) and following i.v. injection of heparin (1000 UI). The adequacy of the anesthesia was tested by the absence of reaction to pinching of the plantar surface. Next, the ascending aorta was cannulated and the heart was subjected to retrograde perfusion with Krebs-Henseleit buffer (115 mM NaCl, 4.6 mM KCl, 1.2 mM KH_2_PO_4_, 1.2 mM MgSO_4_, 2.5 mM CaCl_2_, 25 mM NaHCO_3_ and 11 mM glucose) equilibrated with 95% oxygen and 5% carbon dioxide to a pH of 7.3–7.4. Perfusion was initiated in a non-recirculating Langendorff heart perfusion apparatus at a constant flow rate of 6–8 ml/min to provide a basal perfusion pressure of approximately 70 mmHg. Both the perfusion solution and the heart were maintained at 37°C. Coronary perfusion pressure was measured through a lateral connection in the perfusion cannula and left ventricular pressure was measured using a latex balloon inflated to a diastolic pressure of 5–10 mmHg, both connected to Statham transducers (Statham Instruments, Los Angeles, California) (Figure 1). Left ventricular developed pressure (systolic left ventricular pressure minus diastolic left ventricular pressure), the first derivative of the left ventricular pressure curve (dP/dt) and heart rate were calculated from the left ventricular pressure curve. These parameters were recorded on a computer using Chart 5 v5.4.1 software and the PowerLab/8SP data acquisition system (ADInstruments, Colorado Springs, Colorado).

After a 15 min equilibration period with constant flow perfusion, the hearts were exposed to global zero-flow ischemia for 30 min and reperfused for 15 min at the same flow rate used before ischemia. The duration of ischemia and reperfusion were chosen on the basis of previous studies demonstrating decreases in the endothelium-dependent coronary relaxation without alteration of endothelium-independent coronary relaxation [24], [25]. The control hearts were perfused during a similar total time (60 min) at constant flow without ischemia. After I/R or perfusion during 60 min the coronary vasoconstriction to angiotensin II or the vasodilatation to bradykinin was recorded. Angiotensin II was injected into the perfusion cannula with an infusion pump over 3 min at a constant rate to reach a final concentration of 10^−11^–10^−7^ M. The relaxation to bradykinin was recorded after precontracting the coronary arteries with the thromboxane A_2_ analogue U46619. First, 10^−8^ M U46619 was added to the perfusion solution and the concentration was increased progressively until a contractile tone of ∼120–140 mmHg was obtained. The concentrations of U46619 required to achieve this effect were 1×10^−8^ to 3×10^−8^ M in control conditions and 5×10^−8^ to 2×10^−7^ M after I/R. When the contractile tone reached a stable level, bradykinin was injected into the perfusion cannula over 2 min at a constant rate to reach a final concentration of 10^−9^–10^−6^ M. As the experiments were performed at a constant flow rate, the coronary perfusion pressure provides a measure of the perfusion resistance and characterizes the contraction or relaxation of the coronary arteries.

### Tissue Homogenization and Protein Quantification

Heart tissue was homogenized in 500 µl of radioimmunoprecipitation assay lysis buffer with an EDTA-free protease inhibitor cocktail (Roche Diagnostics, Mannheim, Germany). After homogenization, samples were centrifuged at 14,000 rpm for 20 min at 4°C. Supernatants were transferred to a new tube and protein concentration was estimated by Bradford protein assay.

### Immunoblotting

In each assay the same amount of protein was loaded in all wells (75 µg) and resolving gels with different amount of SDS-acrylamide gels (8–12%) were used depending on the molecular weight of the protein. After electrophoresis proteins were transferred to polyvinylidine difluoride (PVDF) membranes (Bio-Rad) and transfer efficiency was determined by Ponceau red dyeing. Filters were then blocked with Tris-buffered saline (TBS) containing 5% (w/v) non-fat dried milk and incubated with the appropriate primary antibody; caspase-3 (Cell Signalling), caspase-6 (Medical Biological Laboratories), caspase-8 (Neomarkers), Bcl-2 (Thermo Scientific), Hsp-70(Stressgen Bioreagents), iNOS (BD Biosciences), COX-2 (Cell Signalling). Membranes were subsequently washed and incubated with the corresponding secondary antibody conjugated with peroxidase (1∶2000; Pierce, Rockford, IL, USA). Bound peroxidase activity was visualized by chemiluminescence and quantified by densitometry using BioRad Molecular Imager ChemiDoc XRS System. All blots were rehybridized with β-tubulin (Sigma-Aldrich) to normalize each sample for gel-loading variability. All data are normalized to control values on each gel.

### RNA Preparation and Purification and Quantitative Real-time PCR

Total RNA was extracted from the myocardium according to the Tri-Reagent protocol [26]. cDNA was then synthesized from 1 µg of total RNA using a high capacity cDNA reverse transcription kit (Applied Biosystems, Foster City, CA, USA).

### Quantitative Real-time PCR

Angiotensinogen, angiotensin II receptor 1a (*AGTRa*), angiotensin II receptor 2 (*AGTR2*) and pro-renin receptor (*ATP6AP2*) mRNAs were assessed in heart samples by quantitative real-time PCR. Quantitative real-time PCR was performed by using assay-on-demand kits (Applied Biosystems) for each gene: Angiotensinogen (Rn00593114m1), *AGTRa* (Rn02758772s1), *AGTR2* (Rn00560677s1) and *ATP6AP2* (Rn01430718m1). TaqMan Universal PCR Master Mix (Applied Biosystems) was used for amplification according to the manufacturer’s protocol in a Step One machine (Applied Biosystems). Values were normalized to the housekeeping gene 18S (Rn01428915). According to manufacturer’s guidelines, the ΔΔCT method was used to determine relative expression levels. Statistics were performed using ΔΔCT values [27].

### Statistical Analysis

Values are expressed as the mean (± SEM), and compared before and after I/R in rats from control or reduced litters by two way ANOVA. A p value of <0.05 was considered significant.

### Drugs and Chemicals

The following substances were all obtained from Sigma (Tres Cantos, Madrid, Spain): Angiotensin II acetate; bradykinin acetate and 9,11-dideoxy-1a,9a-epoxymethanoprostaglandin F_2α_ (U46619).

## Results

### Body Weight, Fat Mass, Leptin and Angiotensin II Serum Levels

Rats raised in small litters had increased body weight and leptin serum levels at weaning (P<0.001 for both, Table 1), as well as increased epidydimal and subcutaneous fat weights (P<0.001 for both, Table 1) compared to rats raised in control litters. On the contrary angiotensin II serum levels were unchanged between control and overfed rats (Table 1).

**Table 1:** Body weight, epidydimal fat weight, subcutaneous fat weight, leptin and angiotensin II serum levels in rats raised in litters of 12 pups/mother (L12) and rats raised in litters of 3 pups/mother (L3).

	CONTROL	OVERFED
Body weight (g)	45.7±1 (n = 34)	60.7±0.9*** (n = 23)
Epididymal fat (mg)	65.3±3.5 (n = 34)	154.4±8.8*** (n = 23)
Subcutaneous fat (mg)	289±14 (n = 34)	710±36*** (n = 23)
Leptin (ng/ml)	2.4±0.2 (n = 12)	6.7±0.6*** (n = 12)
Angiotensin II(ng/ml)	3.98±0.05 (n = 12)	3.98±0.02 (n = 12)

Data are represented as mean ± SEM.

*** P<0.001 vs L12.

### Haemodynamic Parameters in the Perfused Hearts

Before I/R coronary in the perfused rats, coronary perfusion pressure, maximal dP/dt and heart rate were similar in the rats from control or overfed groups, but left developed intraventricular pressure was significantly lower in the hearts of the rats from the reduced litters (P<0.01,Table 2).

**Table 2:** Hemodynamic values in perfused hearts from control (L12) or overfed (L3) rats before and after 30 min of ischemia and 15 min of reperfusion (I/R).

	Coronary perfusion pressure (mmHg)	Left intraventricular developed pressure (mmHg)	dP/dt (mmHg/s)
CONTROL (n = 19)	72±2	106±14	2415±317
CONTROL+I/R (n = 15)	66±5	40±11#	909±255#
OVERFED (n = 13)	72±2	49±10*	1430±262
OVERFED+I/R (n = 10)	68±6	33±9	839±234

Data are represented as means ±SEM. n = number of hearts.

* (P<0.01). L12 vs. L3.

# (P<0.01) I/R vs. control.

Ischemia-reperfusion induced a significant decrease in left ventricular developed pressure and dP/dt in hearts from control rats (P<0.01) but not in hearts from overfed rats.

### Coronary Vasoconstriction to Angiotensin II

**Figure 2:**
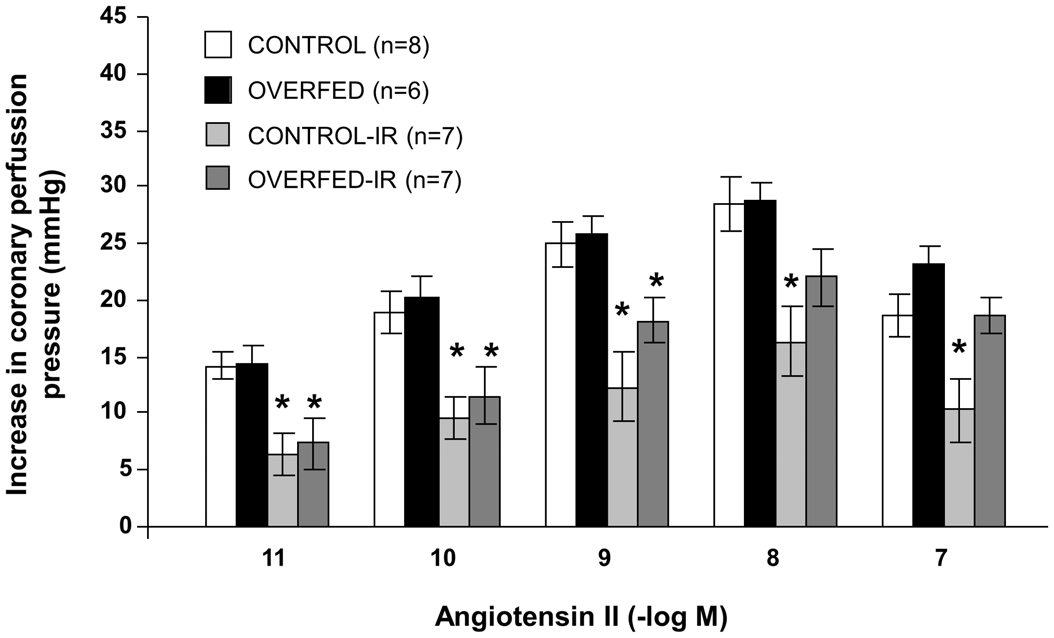
Coronary vasoconstriction to angiotensin II (10^−11^–10^−7^ M) in perfused hearts from control or reduced (overfed) litters, with or without 30 min of ischemia and 15 min of reperfusion (I/R). *P<0.01 I/R vs. control. Values are represented as mean ±S.E.M. n number of hearts.

Injection of angiotensin II into the coronary circulation in the perfused hearts induced concentration-dependent increases of the coronary perfusion pressure (Figure 2). The vasoconstriction to angiotensin II was similar in the hearts from control and overfed rats before ischemia reperfusion. However, after I/R, the vasoconstriction to angiotensin II was reduced in both experimental groups, with the percentage of reduction being significantly smaller in the hearts from overfed rats (% reduction = 40±14, 37±9, 24±4, 15±8 P<0.05, 10±6 P<0.05, for angiotensin II 10^−11^, 10^−10^, 10^−9^, 10^−8^ and 10^−7^ M, respectively) compared to control litters (% reduction = 60±9, 45±10, 46±10, 42±6, 41±9).

### Coronary Vasodilatation to Bradykinin

**Figure 3:**
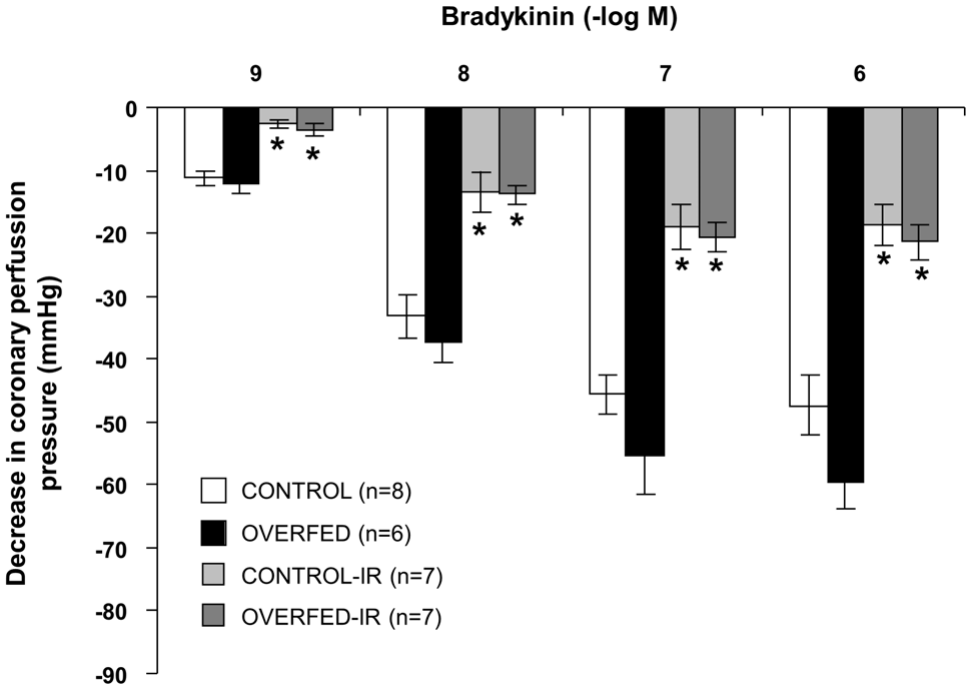
Coronary vasodilatation to bradykinin (10^−9^–10^−6^ M) after precontraction with U46619 in perfused hearts from control or reduced (overfed) litters, with or without 30 min of ischemia and 15 min of reperfusion (IR). *P<0.01 I/R vs. control. Values are represented as mean ±S.E.M. n number of hearts.

The coronary contraction induced with U46619 was similar in control (123±4 mmHg before and 125±6 mmHg after I/R) and overfed (123±3 mmHg before and 123±5 mmHg after I/R) rats. After precontraction of the coronary circulation with U46619, injection of bradykinin induced a significant reduction in the coronary perfusion pressure (Figure 3). This effect of bradykinin was similar in the hearts from overfed and control rats, and was similarly reduced after I/R in both experimental groups.

### Angiotensinogen, *AGTRa*, *AGTR2* and *ATP6AP2* Gene Expression

**Figure 4:**
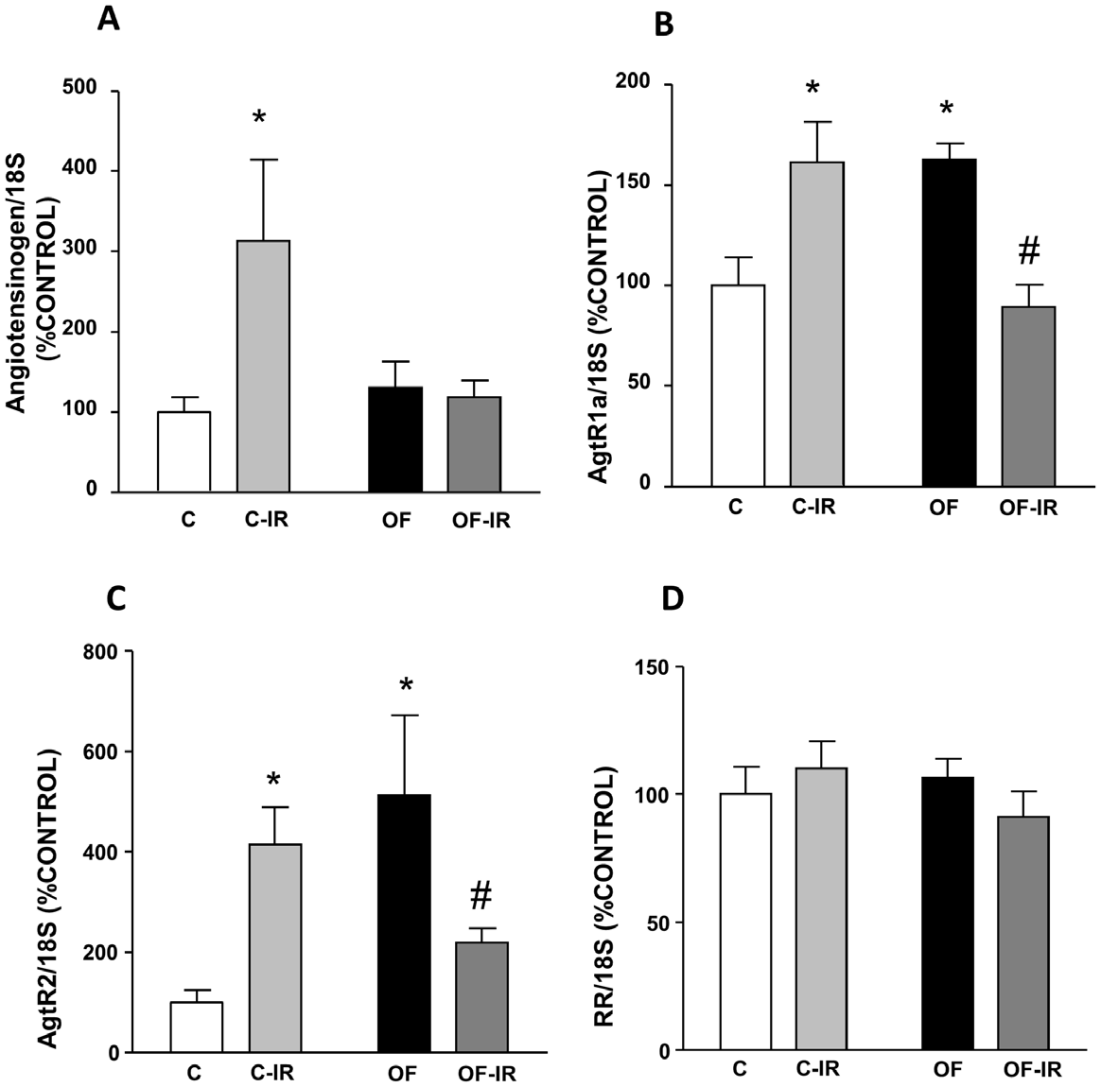
Gene expression of angiotensinogen (A), angiotensin receptor type 1a (*AGTRa*, (B)), angiotensin receptor type 2 (*AGTR2*, (C)) and pro-renin receptor (*ATP6AP2*, (D)) in the myocardium of control and overfed (overfed) rats subjected or not to 30 min of ischemia and 15 min of reperfusion (IR). Values are represented as mean ±S.E.M (n = 6/group).*P<0.05 vs control; #P<0.05 vs control-IR.

Angiotensinogen gene expression was similar in the hearts of control and overfed rats, and it was increased after I/R in the hearts of control (P<0.05) but not in overfed rats (Figure 4A).

*AGTRa* and *AGTR2* gene expressions were up-regulated in the myocardium of overfed rats compared with controls (P<0.05). After I/R, expression of both *AGTRa* and *AGTR2* increased in control but decreased in overfed rats (P<0.05 for both, Figures 4B and 4C,).

*ATP6AP2* was unchanged in response to both early overnutrition or I/R (Figure 4D).

### Apoptotic Markers in the Myocardium

**Figure 5:**
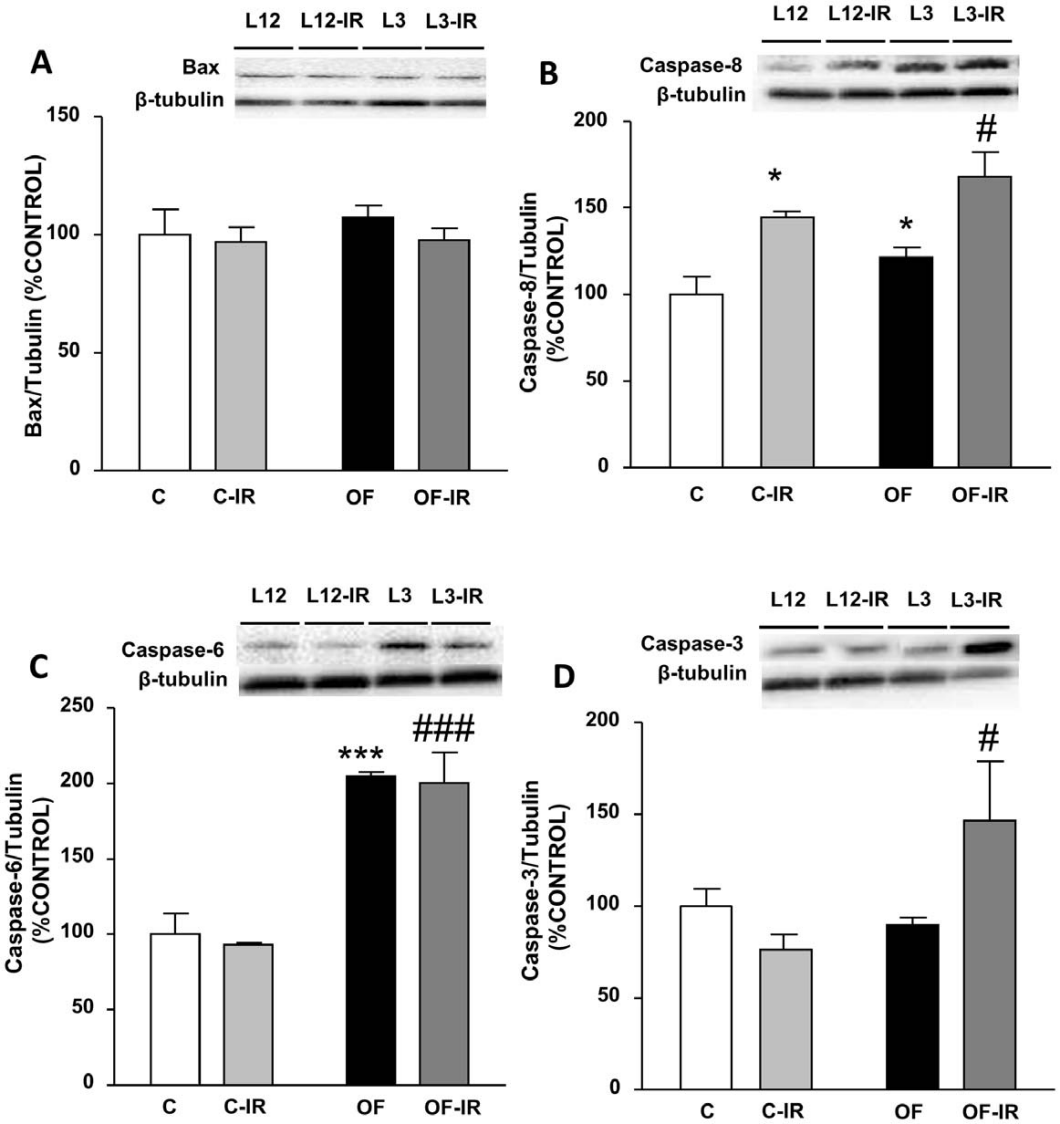
Levels of Bcl-2–associated X protein (Bax, (A)), caspase-8 (B), caspase-6 (C) and caspase-3 (D) in the myocardium of control and overfed (overfed) rats subjected or not to 30 min of ischemia and 15 min of reperfusion (IR). Values are represented as mean ±S.E.M (n = 4–6/group). *P<0.05 vs control; ***P<0.001 vs control; #P<0.05 vs control-IR; ###P<0.001 vs control-IR.

Neither litter reduction nor I/R induced a significant effect in Bax levels in the myocardium (Figure 5A). However, the content of the activator caspase-8 in the myocardium was significantly increased in response to both litter reduction and I/R (P<0.05 for both, Figure 5B). Early overnutrition also had an impact on caspase-3 content in the heart as overfed rats with I/R had increased levels of this proapototic protein compared to control-IR (P<0.05, Figure 5C). In addition litter reduction also increased the myocardic levels of caspase-6 (P<0.001) with I/R having no effect (Figure 5D).

### Anti-apoptotic Markers in the Myocardium

**Figure 6:**
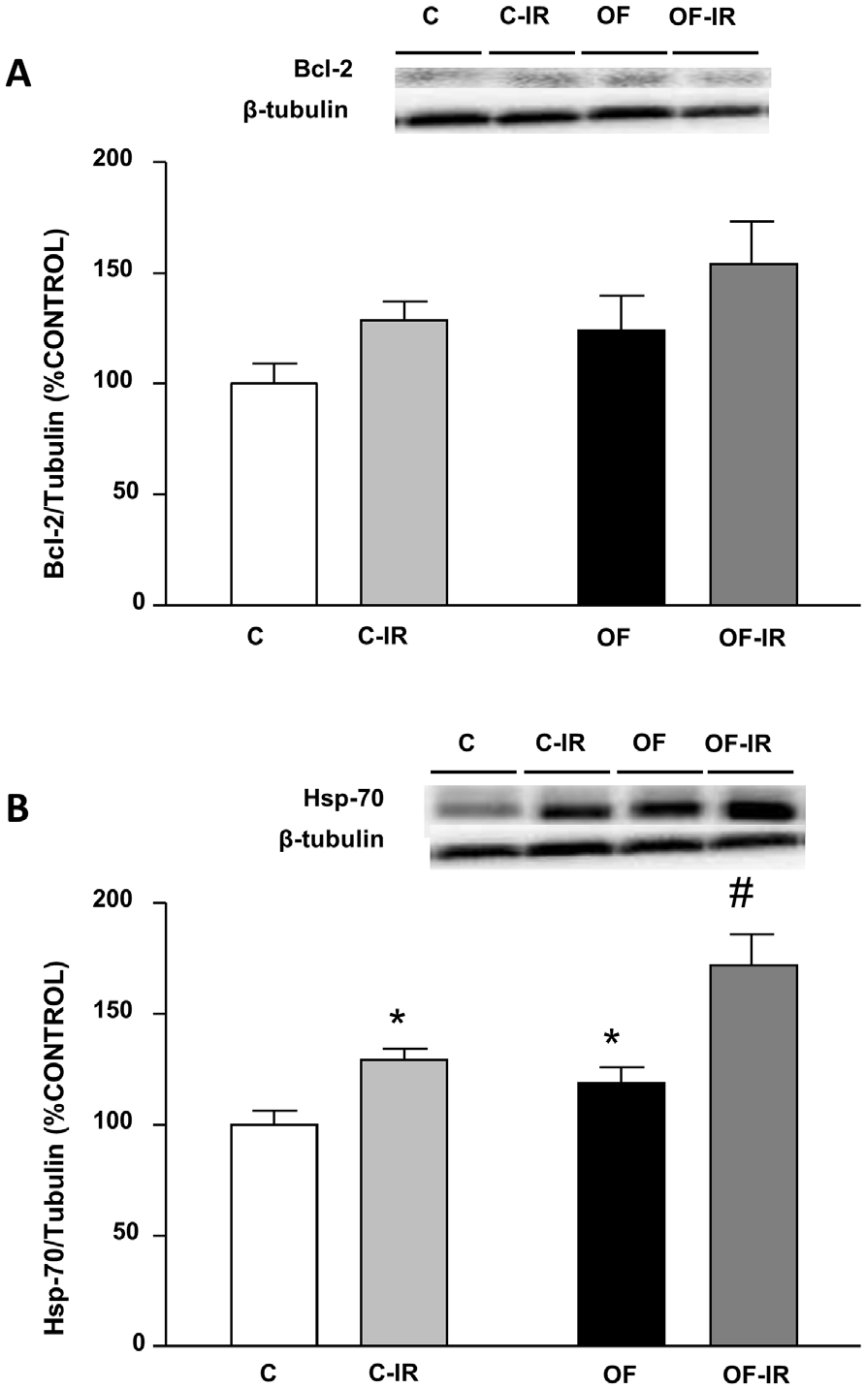
Levels of B-cell lymphoma 2 (Bcl-2, (A)) and heat-shock protein 70 (Hsp-70,(B)) in the myocardium of control and overfed (overfed) rats subjected or not to 30 min of ischemia and 15 min of reperfusion (IR). Values are represented as mean ±S.E.M (n = 4–6/group). *P<0.05 vs control; #P<0.05 vs control-IR.

Bcl-2 levels were unchanged in response to both litter reduction and I/R (Figure 6A). On the contrary Hsp-70 levels were increased in the heart in response to both early overnutrition and I/R (P<0.01 and P<0.001 respectively, Figure 6B), with the levels of this anti-apoptotic protein being greater in overfed-IR rats than in control-IR (P<0.01).

### Inflammatory Markers in the Myocardium

**Figure 7:**
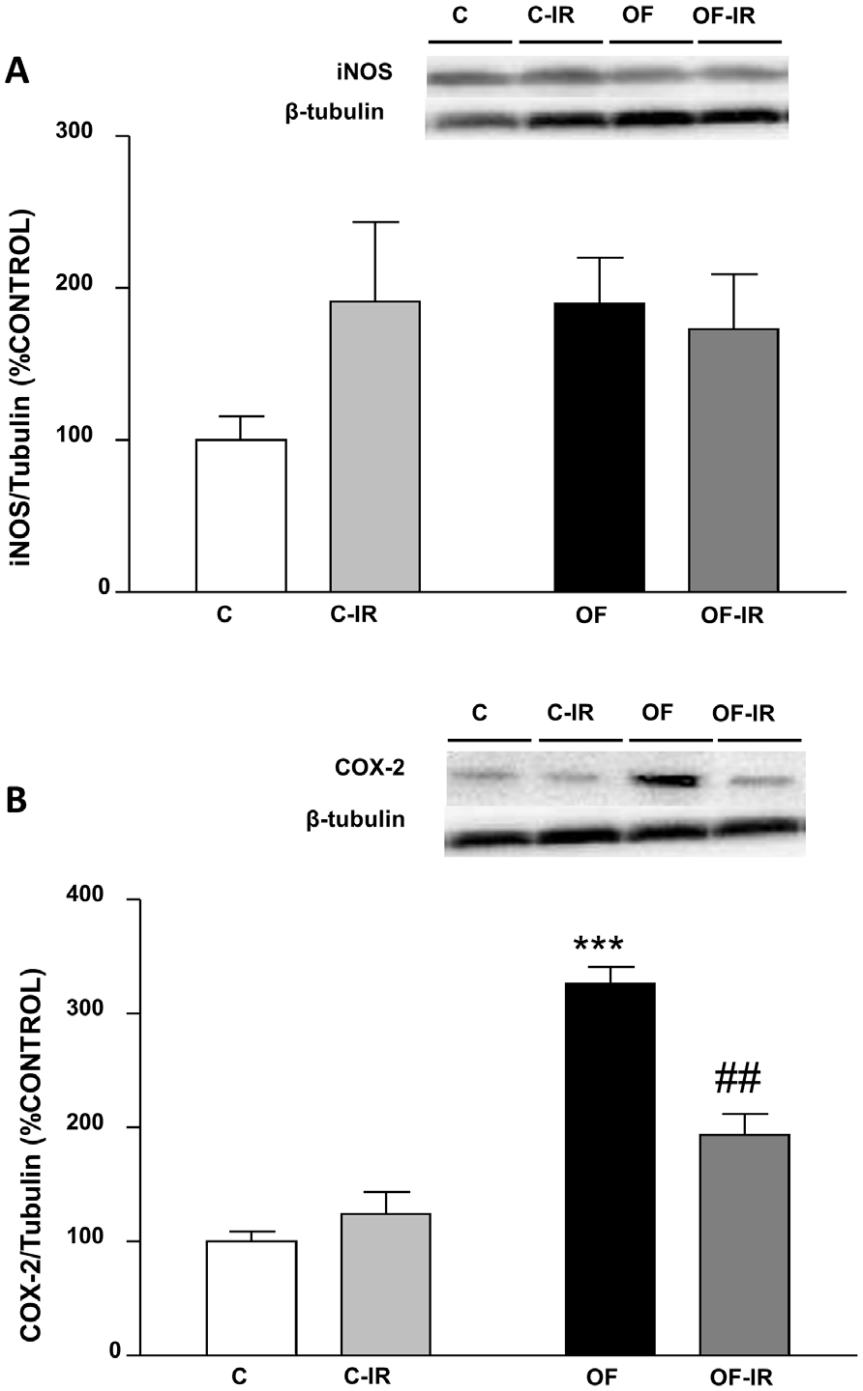
Levels of inducible nitric oxide synthase (iNOS,(A)) and cyclooxigenase-2 (COX-2, (B)) in the myocardium of control and overfed (overfed) rats subjected or not to 30 min of ischemia and 15 min of reperfusion (IR). Values are represented as mean ±S.E.M (n = 4–6/group). ***P<0.001 vs control; ##P<0.01 vs control-IR.

iNOS content in the heart was unchanged in response to both early overfeeding and I/R. COX-2 levels were increased in the heart of overfed rats compared to controls (P<0.001, Figure 7A). I/R did not modify COX-2 levels in the heart of control rats but it decreased the levels of this protein in the heart of overfed rats (P<0.01, Figure 6B).

## Discussion

In this study, we have used an experimental model of early overnutrition in rats by litter reduction in order to assess the effects of early overweight on cardiac function. As previously described, litter reduction increased food intake, and resulted in a higher weight gain and fat mass compared with control litters [28], [21], [29], [30]. This correlated with higher plasma levels of leptin in overfed rats. These alterations may be due, at least in part, to impaired response of neurons in arcuate and ventromedial hypothalamic nuclei to the anorexigenic effects of leptin and insulin [23], [31]as well as to altered metabolic activity of adipose tissue [32].

Previous studies had reported cardiovascular alterations in early overfed rats such an increase in blood pressure [32]and cardiac fibrosis [8]. However, to our knowledge this is the first study showing the deleterious effects of early overnutrition on cardiac function. These alterations could be due, at least in part, to changes in cardiomyocytes maturation as it is reported that this process in the rat takes place during late prenatal or early postnatal life [33]. In addition different perinatal insults, such as poor nutrition, hypoxia and endocrine stress are reported to alter this process inducing an alteration of the number of cells in the myocardium [34].

The perfused hearts from overfed rats showed reduced left ventricular developed pressure, which may be due to impaired myocardial contractility in those hearts. This myocardial impairment was accompanied by increased expression of apoptosis markers in the hearts of overfed rats. However it is interesting that antiapoptotic markers were also increased in this condition, which may constitute an attempt of compensation by myocardial cells. Expression of COX-2 was also increased in the hearts of early overfed rats. This enzyme isoform is induced in inflammatory states, and therefore may be considered an inflammation marker. This apoptotic and inflammatory condition in hearts of overfed rats may damage myocardial cells and result in reduced contractility. In obese patients, increased cardiac output and systolic volume is usually observed, and this increase may be related to the higher blood flow needed as a result of higher body mass. However, when the difference in body weight is taken into account, obese subjects present reduced cardiac index and reduced myocardial contractility [35], [36]. These alterations could be due to increased production of free radicals, inflammatory mediators and apoptotic markers in the heart [37]. Indeed, in the heart of Zucker obese rats, an increase in the levels of apoptotic markers such as ceramide and inflammatory markers such as iNOS has been found [38].

The model of early overnutrition used in the present study may reproduce, at least in part, the effects of childhood obesity. There is evidence that heart alterations due to obesity may begin during childhood. It has been reported that in obese children, although contractile ventricular function is usually preserved, there is already an increase in the index of left ventricular mass [39]. Likewise in obese adolescents, systolic ventricular function may be preserved but diastolic function may present evidence of impairment, which is associated by exercise intolerance [40]. These alterations could be explained, at least in part, by the effect of nutritional conditions on the development of the organs, as it has been reported that perinatal ambient has an important effect on the development of heart or kidney modifying the processes of apoptosis and cell survival [41].

Angiotensin may also be involved in the effects of early overnutrition in the heart. Indeed, early overnutrition is accompanied by hyperleptinemia and this hormone is reported to inhibit angiotensin II-induce vasoconstriction in vitro via a nitric oxide-dependent mechanism [42]. In addition, angiotensin may mediate inflammation and oxidative stress [18], which can lead to apoptosis [19], and these effects may be mediated by *AGTRa* and/or *AGTR2* [43], [44]. We have found in the present study that the expression of *AGTRa* and *AGTR2* was increased in the hearts of overfed rats. This partly agrees with studies finding an increase of *AGTRa* in the kidney [45], or of *AGTR2* in the hearts [46]of obese rats. This overexpression of angiotensin receptors may result in hyperactivity of the angiotensin intracellular pathways, resulting in increased oxidative stress and/or apoptosis and inflammation. Although *AGTR2* are reported to have protective effect in the heart [45], [47], [48], these receptors are also reported to cause cardiac impairment [49], [50], [51], [52], [53]. Therefore the increase in this subtype found in our study may contribute to the reduced contractility in hearts from overfed rats or it may be a compensatory mechanism. Although angiotensin receptors were increased in the hearts of overfed rats, the coronary vasoconstriction to angiotensin II was not modified by early overnutrition. This discrepancy may be due to the fact that *AGTRa* and *AGTR2* have opposite effects on vasomotor responses [54]. As both subtypes are increased in overfed rats, their effects may cancel each other with the final response not being modified.

The deletereous effect of early overnutrition in cardiac contractility could also be related to alterations in baroreflex response due to increased plasma leptin levels. Indeed it has been reported that hyperleptinemia in early stages of development induces persistent sympathoexcitatory hyperresponsiveness with this fact possible mediating an early debut of hypertension [55].

Apoptosis and/or angiotensin pathway activation, in addition to possibly mediate the impairment of cardiac function by overfeeding, may also be involved in the deleterious effect of I/R in the heart [56]. Ischemia-reperfusion induced a reduction in myocardial contractility in rats from control litters. The reduction in myocardial contractility was assessed by developed left intraventricular pressure. In addition, vasoconstriction to angiotensin II and bradykinin induced endothelium-dependent relaxation were also decreased in response to I/R, suggesting that this condition causes damage in the myocardiocytes, vascular smooth muscle and endothelial cells, respectively [24]. These deleterious effects of I/R in the heart were correlated with increased expression of apoptotic markers. Angiotensin system may also be involved, as I/R increased the expression of angiotensinogen and of angiotensin *AGTRa* nd *AGTR2* in control rats.

However, in the hearts of early overfed rats, the deleterious effects of I/R were less marked than in control rats. Indeed, in overfed rats I/R did not significantly reduce myocardial contractility and the reduction in the coronary vasoconstriction to angiotensin II was less marked than in control rats. This may be related to the phenomenon called “the obesity paradox”, by which excess bodyweight may have a protective effect in cardiovascular disease [57]. Several hypothesis have been proposed to account for this phenomenon, like varying levels of tumor necrosis factor α (TNFα) [58]or B-type natriuretic peptide (BNP) [59]. In the present study, the hearts of overfed rats presented a marked increase in the expression of antiapoptotic markers such as Bcl-2 and Hsp-70. Also, in these rats I/R did not increase angiotensinogen gene expression in the heart and expression of angiotensin receptors was not only not increased but reduced after I/R. These changes may reduce I/R-induced myocardial and vascular damage. It may be hypothesized that chronic activation of apoptosis and angiotensin system in overfed rats could induce long-term compensatory mechanisms that may reduce the impairment of myocardial contractility during I/R.

In conclusion, both overfeeding and I/R impairs cardiac and coronary function due, at least in part, to activation of angiotensin pathway. However, overfeeding may reduce some of the harmful effects of I/R, which may be due to activation of compensatory mechanisms.
